# Severe pain management in the emergency department: patient pathway as a new factor associated with IV morphine prescription

**DOI:** 10.3389/fpubh.2024.1352833

**Published:** 2024-02-22

**Authors:** Virginie E. Lvovschi, Florence Carrouel, Karl Hermann, Frédéric Lapostolle, Luc-Marie Joly, Marie-Pierre Tavolacci

**Affiliations:** ^1^Emergency Department, Hôpital Edouard Herriot, Hospices Civils de Lyon, Lyon, France; ^2^Laboratory “Research on Healthcare Performance” (RESHAPE), INSERM U1290, Université Claude Bernard Lyon 1, Lyon, France; ^3^Laboratory “Health, Systemic, Process” (P2S), UR4129, University Claude Bernard Lyon 1, University of Lyon, Lyon, France; ^4^Rouen University Hospital, CIC-CRB 1404, Rouen, France; ^5^SAMU 93, UF Research and Teaching quality, Avicenne Hospital-APHP, Bobigny, France; ^6^INSERM U942, Sorbonne Paris Cité, Paris 13 University, Paris, France; ^7^Emergency Department, Rouen University Hospital, Rouen, France; ^8^Univ Rouen Normandie, UMR1073 ADEN, Rouen, France

**Keywords:** severe pain, oligoanalgesia, intravenous morphine titration, pain management, emergency department, opioids, care pathway, outpatient

## Abstract

**Background:**

Across the world, 25–29% of the population suffer from pain. Pain is the most frequent reason for an emergency department (ED) visit. This symptom is involved in approximately 70% of all ED visits. The effective management of acute pain with adequate analgesia remains a challenge, especially for severe pain. Intravenous (IV) morphine protocols are currently indicated. These protocols are based on patient-reported scores, most often after an immediate evaluation of pain intensity at triage. However, they are not systematically prescribed. This aspect could be explained by the fact that physicians individualize opioid pain management for each patient and each care pathway to determine the best benefit–risk balance. Few data are available regarding bedside organizational factors involved in this phenomenon.

**Objective:**

This study aimed to analyze the organizational factors associated with no IV morphine prescription in a standardized context of opioid management in a tertiary-care ED.

**Methods:**

A 3-month prospective study with a case–control design was conducted in a French university hospital ED. This study focused on factors associated with protocol avoidance despite a visual analog scale (VAS) ≥60 or a numeric rating scale (NRS) ≥6 at triage. Pain components, physician characteristics, patient epidemiologic characteristics, and care pathways were considered. Qualitative variables (percentages) were compared using Fisher’s exact test or the chi-squared tests. Student’s t-test was used to compare continuous variables. The results were expressed as means with their standard deviation (SD). Factors associated with morphine avoidance were identified by logistic regression.

**Results:**

A total of 204 patients were included in this study. A total of 46 cases (IV morphine) and 158 controls (IV morphine avoidance) were compared (3:1 ratio). Pain patterns and patient’s epidemiologic characteristics were not associated with an IV morphine prescription. Regarding NRS intervals, the results suggest a practice disconnected from the patient’s initial self-report. IV morphine avoidance was significantly associated with care pathways. A significant difference between the IV morphine group and the IV morphine avoidance group was observed for “self-referral” [adjusted odds ratio (aOR): 5.11, 95% CIs: 2.32–12.18, *p* < 0.0001] and patients’ trajectories (Fisher’s exact test; *p* < 0.0001), suggesting IV morphine avoidance in ambulatory pathways. In addition, “junior physician grade” was associated with IV morphine avoidance (aOR: 2.35, 95% CIs: 1.09–5.25, *p* = 0.03), but physician gender was not.

**Conclusion:**

This bedside case–control study highlights that IV morphine avoidance in the ED could be associated with ambulatory pathways. It confirms the decreased choice of “NRS-only” IV morphine protocols for all patients, including non-trauma patterns. Modern pain education should propose new tools for pain evaluation that integrate the heterogeneity of ED pathways.

## 1 Introduction

The World Health Organization (WHO) and Human Rights Watch declared that pain management was a human right (1). Across the world, 25–29% of the population suffers from pain (1). Pain is the most frequent symptom in the emergency department (ED). Pain is present in approximately 70% of all ED visits (2). Acute pain is an extremely complex sensory experience involving affective and motivational components following afferent inputs in the neurological system (3). The acute pain experience is a difficult outcome to predict because it can be mediated by several psychological and psychosocial factors. Thus, the effective management of acute pain and providing appropriate analgesia pose significant challenges. In fact, pain management is associated with major short- and long-term outcomes, such as sleep quality, maintenance of physical functions, and quality of life. The effective management of acute pain is essential to prevent the development of longer-term chronic pain (4–6). In addition, shorter delays in analgesic administration are associated with shorter ED length stays (7), which is associated with improved quality of life (8), reduced morbidity, and reduced mortality (9, 10).

A wide range of pharmacologic treatments are available for the management of acute pain. Analgesic options include both opioid and non-opioid treatments with a variety of formulations and routes of administration (11). As a result, practices in the use of opioids and other analgesics vary widely across the world (12–14) and particularly in Europe (15).

Determining the most appropriate drug for patients is a decision-making process that depends on the context (ED or pre-hospital). In addition, caregivers involved in pain management and treatment administration (e.g., nurses and paramedics), such as emergency physicians, vary in terms of education, training, and job specification. These elements impact their ability/authority to provide analgesics (16–18). In summary, the factors that determine the administration of analgesics are the ability of healthcare personnel to prescribe and dispense various analgesics, the intensity of pain quantified by the patient, and the recommendations on the class of analgesics according to scientific authorities such as the WHO (19).

Opioids are essential for the treatment of acute pain but are involved in numerous toxic risks, particularly nausea, vomiting, pruritus, urinary retention, constipation, ileus, sedation, delirium, and respiratory depression (20). These opioid-related adverse effects lead to increased hospital stays and economic burden (21, 22). Repeated exposure to opioids leads to μ-receptor desensitization and tolerance, which contributes to opioid use disorder and opioid withdrawal syndrome (23, 24). In addition, the opioid crisis and its consequences raise questions about the more targeted use of these analgesics (25, 26). Because severe pain can lead to serious outcomes (27–29), morphine remains a potent opioid commonly used for this indication in pre-hospital and ED settings (30). Morphine can be delivered via intravenous (IV), subcutaneous, intramuscular, and oral routes (31, 32). Numerous international guidelines recommend IV morphine as the standard of care for the effective management of severe acute pain in emergency settings (33–36).

In France, IV morphine titration has been recommended since 2010 for patients presenting to the ED with severe pain assessed by validated pain scales [a visual analog scale (VAS) ≥ 60/100 and a numeric rating scale (NRS) ≥ 6/10] (37). Indeed, morphine has been shown to be safe, feasible, and effective in large populations (38). Standardized protocols based on VAS/NRS at triage have been proposed to improve its implementation and reduce the delay to morphine administration (39–41), including initiation by nurses before physician evaluation (42).

Nevertheless, the management of severe pain remains a major challenge in the ED in France. Emergency physicians do not always follow these recommendations, as evidenced by the low rate of IV titration (≤10%) and the delay in administration (43). This lack of adherence raises questions. Morphine is recognized for its efficacy in the treatment of acute pain (44), it has a good benefit–risk ratio (38), and pain management education programs have insisted on its prescription for years (45–47). Several reasons could explain this non-adherence: (i) non-opioid alternatives are currently validated for specific pain patterns such as uncomplicated renal colic, musculoskeletal conditions, or headache (48–50), (ii) protocols based only on initial VAS/NRS evaluation have been questioned (51), (iii) the need for venous access may be an obstacle, (iv) overcrowding degrades opioid management (52), and (v) the development of strategies to optimize the patient pathway may be necessary.

Therefore, determining the reasons for this non-adherence to recommendations (first-line IV morphine prescription) to improve patient management for acute pain is significantly important. In a previous study, ED physicians reported pain etiology as the main reason for not prescribing IV morphine. No effect of common organizational constraints on IV morphine titration was found (a lack of available nurses, delays in patient installation in the care area, no need for venous access, etc.) (53). In fact, few ED data are available on real-time factors associated with bedside IV morphine avoidance, including organizational issues (43, 54, 55).

This study aimed to identify organizational factors associated with IV morphine avoidance in a standardized context of opioid management in a tertiary ED.

## 2 Materials and methods

### 2.1 Study design

A case–control study represents a common type of observational study designed to investigate factors related to diseases or outcomes (56). Patients are selected based on an outcome, with cases defined as patients with the outcome, and controls defined as patients without the outcome. The proportion of exposed patients is then compared between cases and controls. Prospective case–control studies involve the selection of a group of individuals and their follow-up (during planned health surveillance). Cases are individuals who acquire the disease or condition of interest during the course of the study. Individuals who are not affected by the disease form the control group (57). Exposures precede the outcome chronologically. Contrary to cohort studies, only a subset of patients without the outcome (controls) is analyzed due to research cost constraints. Otherwise, cases and controls are comparable, as in a cohort study.

This case–control study is a prospective bedside study conducted by a team of four researchers, with “real-time” inclusions, assessing the organizational factors of the decision-making process of prescription. This research was designed as a case–control study (58), similar to other opioid-related studies (59). The outcome was “IV morphine administration” after prescription in patients with severe pain in the ED. Exposures included patient and physician characteristics at admission (chronologically prior to exposure). In this specific design, IV morphine was “exposition/disease.” Thus, in this case–control study, subjects were selected based on the outcome, and exposures were factors at the time of ED admission that could be considered by the prescribing physician. The final inclusion of patients by the research technician was performed at the time of discharge from the ED, as the outcome was known for all patients at that time. Consecutive eligible cases and controls were recruited until the target number of cases and controls was reached. Therefore, there was a high rate of inclusion of controls during the first weeks, after which only cases were recruited. This study was carried out in accordance with STROBE guidelines (Supplementary Table S1). The ethics committee of Rouen University Hospital approved this study (Review Board number E2019-27; approval date 4 December 2019), which was performed in accordance with the principles of the Declaration of Helsinki. Written/oral informed consent was obtained from all participating physicians.

### 2.2 Setting

This prospective study was conducted in the ED of a French university hospital (Rouen, Normandy, 110,000 visits annually). This ED is organized into one triage area, one critical care area, and two non-critical care areas (medical vs. surgical), each with its own ambulatory pathway.

### 2.3 Objectives

#### 2.3.1 Primary objective measures

The primary objective of this study was to evaluate whether organizational factors (physicians’ characteristics and care pathways) were associated with adherence to the recommended protocol for the treatment of severe pain in an ED.

#### 2.3.2 Secondary objective measures

The secondary objective of this study was to evaluate whether patient’s epidemiological factors and pain components were associated with adherence to the recommended protocol for the treatment of severe pain in an ED.

### 2.4 Participants

All physicians who treated patients with acute pain in the ED during the period of this study (7 January to 23 March 2019) were eligible and could be interviewed by one of the four researchers regarding the management of patients’ acute pain.

All adult patients presenting to the care areas of ED (excluding critical care) on weekdays between 8 a.m. and 5 p.m. were screened in real-time using our computerized information system in the triage and care areas. Patients identified at triage as having severe pain using the VAS or NRS scales (VAS ≥ 60/100 or NRS ≥ 6/10) were prospectively included.

There were no exclusions related to the home pain management regimen, including major analgesics.

### 2.5 Sample size

According to the low prevalence of the protocol follow-up (6%) observed in an initial 1-month period (53), this study aimed to include 50 cases using a 3:1 case study design. The duration of this prospective study was estimated to be approximately 3 months. This decision was initially based on bedside feasibility. In addition, no clinically relevant factor was considered *a priori* to be predominant. With a type I error rate of 5%, 52 cases (receiving IV morphine) and 156 controls, 60% of patients returning home in the control group, an odds ratio of 3 between returning home and not receiving IV morphine, and a case–control ratio of 1:3, the statistical power would be 80%. This calculation supported our decision to include this number of patients.

### 2.6 Study procedure: pain management for patients with acute severe pain

IV morphine titration is a pharmacological method that involves repeated administration of small IV morphine boluses every 5 min until pain relief (VAS ≤ 30 or NRS ≤ 3). For patients weighing more than 60 kg, each bolus dose is 3 mg. For patients weighing less than 60 kg, each bolus dose is 2 mg. IV morphine titration is interrupted before pain relief in case of excessive sedation (Ramsay Score > 2) (60) or respiratory side effects (respiratory rate < 10/min and/or oximetry <92%).

At the Rouen University Hospital, France, IV morphine titration is a daily practice, a standardized pain management that is automatically proposed in the care areas when a patient’s VAS is ≥60/100 or NRS is ≥6/10 at triage. This practice is based on a locally validated protocol implemented in 2015, which is in line with French recommendations (37). VAS/NRS assessments are systematically performed in the triage area, and the scores are compulsorily recorded in the electronic health records (M-UrQual software v. 7) of our computerized information system (HEO software 8.2; v 8.2; Maincare Solutions, France) of our ED. Immediately after a patient’s transfer to a care area, when physicians and nurses are informed of a high VAS/NRS score, they must confirm the initiation of the protocol. Once the protocol is confirmed, the patient is immediately placed in an examination room, venous access is established, and morphine titration is initiated with the goal of NRS ≤ 3. Other IV modalities or routes of morphine administration (oral, intramuscular, and subcutaneous) are recommended as secondary options. The intranasal route of administration is not used in our ED. First-line care is provided by either junior or senior physicians. Junior physicians are medicine residents (≤5 years of experience), whereas senior physicians are non-residents. At the end of ED care, all patients are managed by senior physicians.

#### 2.6.1 Classification of participants as case or control

Patients who received IV morphine according to the protocol (from prescription to administration) were allocated to the case group (IV morphine), while other patients were assigned to the control group (avoidance of IV morphine). Patients who did not receive IV morphine but who had a first morphine prescription by physicians in the care area were excluded. Conversely, according to the principles of standard care, common analgesics [acetaminophen, non-steroidal anti-inflammatory drugs (NSAIDs), tramadol, nefopam, etc.] and co-analgesics (as antispasmodics) were allowed in both groups, regardless of the time of administration.

#### 2.6.2 Data collection and evaluation criteria

The classic epidemiologic characteristics of patients, pain components, physician characteristics, and patient care pathways were assessed. Data on morphine titration decisions and physician characteristics (grade and sex) were collected by a physician interview conducted by researchers at the patient’s bedside. Electronic health records were analyzed for other parameters. In case of missing data, physicians were questioned by the researcher team in real-time.

Pain components included VAS/NRS scores and four pain patterns based on the diagnosis at discharge: the “traumatic” pattern, the “visceral and urogenital” pattern, the “musculoskeletal” pattern (including spinal disorders), and a final “other non-musculoskeletal medical conditions” pattern (including chest pain and headache). The epidemiologic characteristics of patients included sex and age. Care pathways independently included time of arrival (between 0 a.m. and 11 a.m. or between 0 p.m. and 11 p.m.), admission route (self-referral vs. ambulance), discharge mode (hospitalization vs. discharge home), and patient trajectory (self-referral/discharge home, self-referral/hospitalization, ambulance/discharge home, or ambulance/hospitalization). Physician characteristics relative to first-line physicians included grade (junior or senior), sex, and age. Classification by grade was necessary as junior physicians had their prescriptions reviewed by senior physicians (61) and this study focused on the initial prescription decision.

### 2.7 Statistical analysis

Qualitative variables (percentages) were compared by Fisher’s exact test or the chi-squared test when all expected frequencies of the contingency table were ≥ 5. Continuous variables (means with standard deviation [SD]) were compared using Student’s *t*-test. Factors associated with morphine avoidance were identified by a multivariate logistic regression analysis. Variables included in the multivariate analysis were as follows: sex, admission route, VAS/NRS score, pain pattern, and physician grade. This analysis was not applicable to discharge mode, which was determined by physicians according to the outcome of “morphine exposure.” Adjusted odds ratios (aORs) and their 95% confidence intervals (CIs) were calculated. Variables leading to IV morphine prescription did not correspond to “indication bias” but rather to “indication effects,” despite differences in characteristics.

Interaction terms (i.e., physician grade*admission route, physician grade*NRS score, physician grade*pain pattern, and physician grade*mode of discharge) were tested with respect to the behavioral variables included in the logistic regression. The discriminative power of the logistic regression was assessed by the area under the receiver operating characteristic (ROC) curve and by the McFadden pseudo-R^2^. The calibration of the logistic regression was assessed by the Hosmer–Lemeshow test. Eight missing data points on the admission route were simply imputed by the modal class of the subgroup of patients with the same discharge mode (home or hospitalization); self-referral for home discharge; and ambulance for hospitalization. Statistical significance was defined as a value of *p* of <0.05. R (version 4.2, The R Foundation for Statistical Computing, Vienna, Austria) was used for analyses.

## 3 Results

### 3.1 Patient recruitment

**Figure 1:**
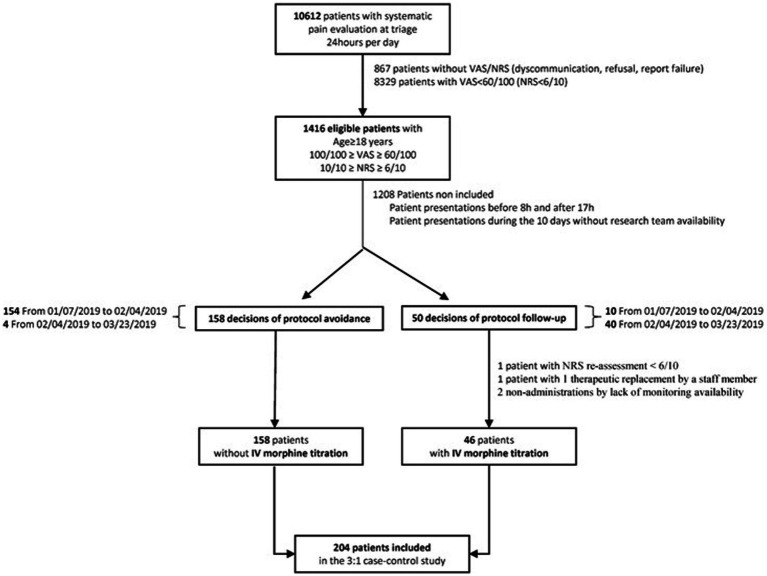
Study flowchart. IV, intravenous; NRS, numeric rating scale; VAS, visual analog scale.

Figure 1 presents the flowchart of the study. The ED received 10,612 patients with systematic pain evaluations at triage. Of these 10,612 patients, 1,416 patients were eligible, and 204 patients were finally included, of which 46 cases were recruited between 7 January and 28 March 2019 and 158 controls between 7 January and 23 March 2019.

Fifty IV morphine titration decisions were considered. No exclusions were related to the alternative routes of morphine administration (including IV modes without titration). A total of 48 patients of the 50 IV morphine prescriptions were maintained. One exclusion was related to a NRS score of <6/10, and one therapeutic replacement resulted in a secondary exclusion. IV morphine was administered in 46 out of 48 patients. Two administrations were canceled due to a lack of monitoring availability. The final morphine administration corresponded to 92% of the initial prescription decision.

### 3.2 Characteristics and pain components of patients treated with or without IV morphine

Patient characteristics, pain levels, and pain patterns are depicted in Table 1. The sex and age of the patients were not significantly different in the IV morphine avoidance group and the IV morphine group: 45.6% vs. 45.7% of male patients, respectively, and a mean age of 45.7 (20.0) years vs. 45.4 (20.2) years.

**Table 1:** Characteristics of patients and pain components according to IV morphine exposure.

	**No IV morphine group (*n* = 158) *n* (%)** ^**a**^	**IV morphine group (*n* = 46) *n* (%)** ^**a**^	***p***	**Adjusted Odds ratio (CI 95%)** ^**b**^	***p***
**Characteristics of patients**					
*Sex*			>0.99		0.87
Male (*n* = 93)	72 (45.6%)	21 (45.7%)		1 (reference)	
Female (*n* = 111)	86 (54.4%)	25 (54.3%)		0.94 (0.43–2.03)	
Age (*n* = 204)	45.8 ± 20	45.4 ± 20.2	0.92		
**Pain components**					
*NRS scores*			0.0004^c^		0.01^c^
10 (*n* = 37)	20 (12.7%)	17 (37.0%)		1 (reference)	
9 (*n* = 24)	18 (11.4%)	6 (13.0%)		2.45 (0.73–8.92)	0.15
8 (*n* = 48)	38 (24.1%)	10 (21.7%)		2.97 (1.06–8.74)	0.038
7 (*n* = 45)	41 (25.9%)	4 (8.7%)		5.76 (1.68–23.81)	0.005
6 (*n* = 50)	41 (25.9%)	9 (19.6%)		3.24 (1.13–9.8)	0.028
*Pain patterns*			0.13		
Non-musculoskeletal (*n* = 89)	74 (46.8%)	15 (32.6%)		1 (reference)	
Visceral and urogenital (*n* = 40)	27 (17.1%)	13 (28.3%)		0.51 (0.19–1.37)	0.18
Musculoskeletal (*n* = 28)	19 (12.0%)	9 (19.6%)		0.54 (0.18–1.69)	0.29
Trauma (*n* = 47)	38 (24.1%)	9 (19.6%)		0.9 (0.32–2.61)	0.84

^a^Except age: mean and standard deviation. Significance is defined as *p* < 0.05. IV, intravenous; NRS, numeric rating scale. ^b^Multivariate model including sex, NRS score, pain pattern, admission route, time of arrival, and physician’s grade. ^c^Test for linear trend.

Regarding pain components, the NRS was the only assessment tool used by the triage nurses. The mean NRS did not differ significantly by the pain pattern (*p* = 0.18). The mean NRS scores were 7.5 (SD 1.4), 7.7 (SD 1.4), 7.8 (SD 1.4), and 8.1 (SD 1.3) for trauma, non-musculoskeletal, musculoskeletal, and visceral and urogenital patterns, respectively. No significant differences were observed between the two groups for pain patterns, whereas significant differences were observed for NRS scores. Most morphine-treated patients had an NRS score of 10 (37.0%), while an NRS score of 6 or 7 was observed in IV morphine avoidance patients (25.9% for both scores). In addition, the multivariate analysis suggested that a NRS score lower than 7 (aOR 5.76 95%CI 1.68–23.81) could be associated with IV morphine avoidance (*p* = 0.01).

### 3.3 Organizational factors determining IV morphine avoidance in patients admitted to the emergency department

The univariate analysis and multivariate analysis of the factors associated with physician characteristics and care pathways are presented in Table 2.

**Table 2:** Characteristics of first-line physicians and care pathways according to IV morphine exposure.

	Non-IV morphine group (*n* = 158) %*	IV morphine group (*n* = 46) %*	***p***	**Adjusted Odds ratio (CI 95%)** ^**a**^	***p***
**Care pathways**					
*Admission route*			<0.0001		<0.0001
Ambulance (*n* = 100)	64 (40.5%)	36 (78.3%)		1 (reference)	
Self-referral (*n* = 104)	94 (59.5%)	10 (21.7%)		5.11 (2.32–12.18)	
*Time of arrival*			0.32		0.17
0 a.m.–11 a.m. (*n* = 111)	83 (52.5%)	28 (60.9%)		1 (reference)	
12 a.m.–11 p.m. (*n* = 93)	75 (47.5%)	18 (39.1%)		1.73 (0.8–3.86)	
*Mode of discharge*			<0.0001		
Hospitalization (*n* = 68)	34 (21.5%)	34 (73.9%)		NA	NA
Discharge home (*n* = 136)	124 (78.5%)	12 (26.1%)		NA	NA
**Characteristics of physicians**					
*Grade*			0.014		0.029
Senior (*n* = 96)	67 (42.4%)	29 (63.0%)		1 (reference)	
Junior (*n* = 108)	91 (57.6%)	17 (37.0%)		2.35 (1.09–5.25)	
*Sex*			0.37		
Male (*n* = 78)	63 (39.9%)	15 (32.6%)			
Female (*n* = 126)	95 (60.1%)	31 (67.4%)			

^a^Multivariate model including sex, NRS score, pain pattern, admission route, time of arrival, and physician’s grade. Interaction: grade of physician *admission route *p* = 0.07/grade of physician *NRS score *p* = 0.26/grade of physician *Pain pattern *p* = 0.14/grade of physician *mode of discharge *p* = 0.58. *Except age: mean and standard deviation. Significance is defined as *p* < 0.05. IV, intravenous; NRS, numeric rating scale; NA, not applicable.

Regarding the care pathways, the time of arrival to the ED was not significantly different between groups (*p* = 0.32), in contrast to the admission route (40.5% of patients arrived by ambulance in the IV morphine avoidance group vs. 78.3% in the IV morphine group). Discharge mode was significantly different (21.5% of patients in the IV morphine avoidance group were hospitalized vs. 73.9% in the IV morphine group). In addition, self-referral was significantly associated with IV morphine avoidance in the multivariate analysis (aOR 5.11, 95% CI 2.32–12.18).

Regarding physician characteristics, senior physicians were more likely to prescribe IV morphine (63.0%) for first-line care. The junior physician grade was significantly associated with IV morphine avoidance (aOR 2.35, 95%CI 1.09–5.25). Patient age, NRS score, pain pattern, self-referral, and discharge mode did not significantly differ by the physician grade. Physicians’ sex did not significantly affect IV morphine avoidance.

The area under the ROC curve of the logistic regression was estimated to be 0.88 (95% CI 0.82–0.93), and the McFadden pseudo-R^2^ was estimated to be 0.20. The Hosmer–Lemeshow test showed no significant calibration flaw (*p* = 0.40).

### 3.4 Rate of morphine administration according to patient trajectory

**Figure 2:**
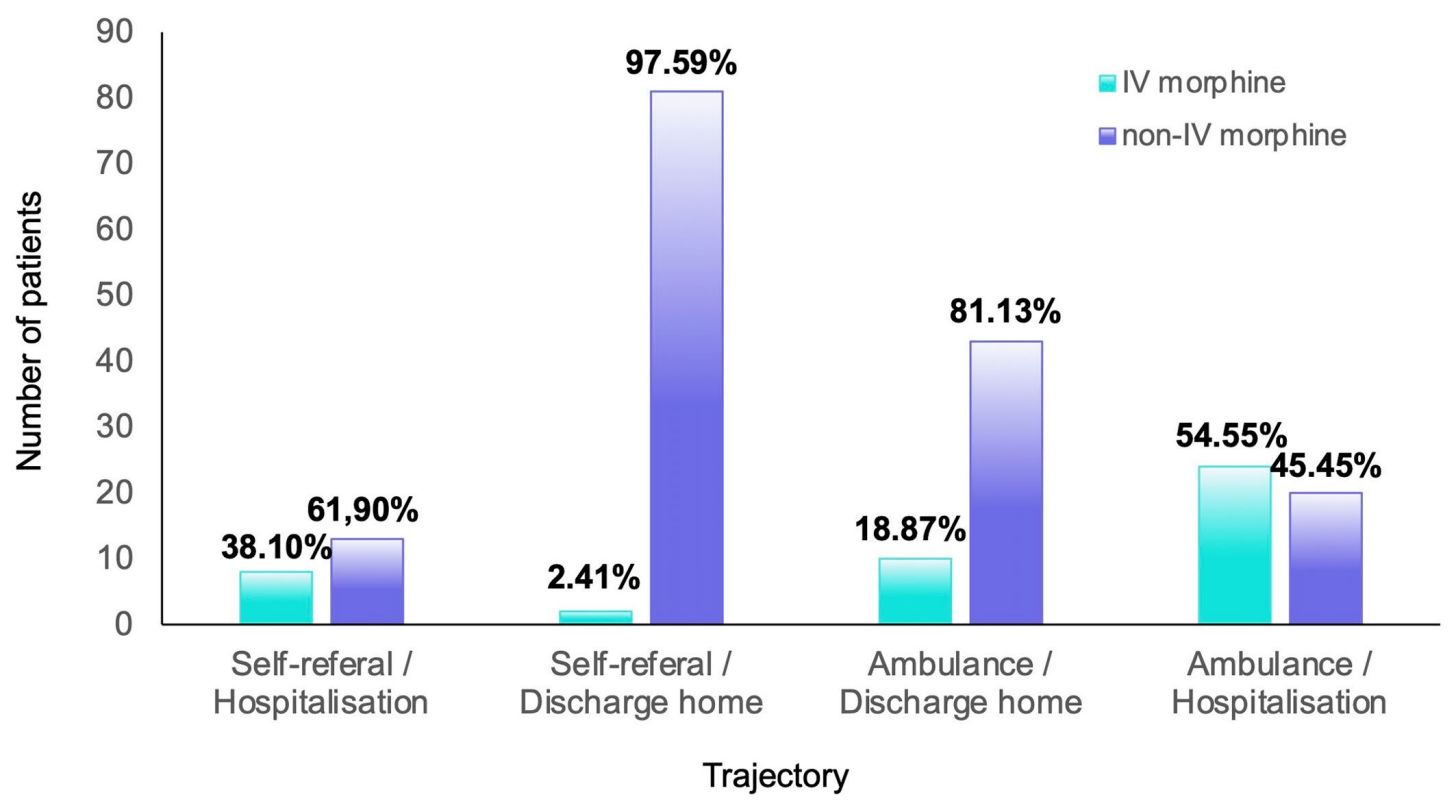
Repartition of patients according to the care trajectory and management. The percentages were calculated according to the total number of patients for each trajectory.

IV morphine distribution according to patient trajectory is detailed in Figure 2 and Table 3. The trajectory was mainly self-referral/discharge home (51.3%) in the IV morphine avoidance group and ambulance/hospitalization (56.5%) in the IV morphine group. Most of the patients whose trajectory ended with discharge home were not managed with an IV morphine protocol. A total of 10 of the 53 patients with an ambulance/discharge home trajectory (18.9%) and 2 of the 83 patients with a self-referral/discharge home trajectory (2.4%) received IV morphine titration. Fisher’s exact test (two-sided) showed a significant difference between the two groups (*p* < 0.0001).

**Table 3:** Distribution of patients according to the care trajectory and management.

	**No IV morphine group *n* (%** ^**a**^ **)**	**IV morphine group *n* (%** ^**a**^ **)**
Self-referral/hospitalization	13 (8.2%)	8 (17.4%)
Self-referral/discharge home	81 (51.3%)	2 (4.3%)
Ambulance/discharge home	43 (27.2%)	10 (21.7%)
Ambulance/hospitalization	21 (13.3%)	26 (56.5%)

^a^The percentage were calculated according to the total number of patients treated with IV morphine or non-IV morphine.

## 4 Discussion

This prospective case–control study was conducted by four researchers at bedside. This study analyzed the prescription of IV morphine (outcome) in 204 patients (46 cases and 158 controls) who presented to a tertiary ED with severe acute pain between 7 January and 28 March 2019. It also aimed to determine factors associated with the avoidance of a standardized IV morphine protocol in pain management. To our knowledge, this is the first report on this topic to analyze the impact of care pathways irrespective of pain typology or intensity. Most authors have focused on traumatological or visceral patterns, arguing that increased severity in these patterns leads to a higher risk of oligoanalgesia (17, 62–64).

This study highlights that the care pathway was a major factor associated with IV morphine titration. First, ambulatory patients received IV morphine titration less frequently than other patients. The self-referral and discharge home pathways were significantly associated with IV morphine avoidance. Data on patient trajectory support this hypothesis. Anticipation of a short length of stay and a lack of available beds may be related to this limitation. Ambulatory pathways could lead to a downregulation of IV morphine prescription. Physicians may initially prescribe non-opioid analgesics, such as NSAIDs or acetaminophen, to shorten the duration of pain management (65). IV morphine titration requires more nurse time than orally administered analgesics. A lack of the availability of safe rooms for titration, sometimes with long delays, could also explain protocol avoidance. In addition, morphine is subject to strict legal control in the EDs of French hospitals (66). This aspect has advantages in the context of the opioid crisis, but it lengthens the prescribing process. The strict control may be an additional barrier to prescribing either consciously or unconsciously. Flow management can also contribute to protocol avoidance. IV morphine titration requires monitoring for ≥2 h after completion. The literature has demonstrated a statistical association between crowding and degradation of pain management, focusing on the delay to first analgesic administration as well as the delay from room placement to first analgesic administration (54). For ambulatory patients, the goal is most likely to avoid room placement before administering analgesia. Moreover, the modern concern of ED physicians is to limit unnecessary venous lines as much as possible. Venous lines unnecessarily impede the patient’s free flow within the ED and cause increased delays in x-ray investigations. The venous line risk–benefit ratio remains unclear (thrombosis,…) (67–71). Another hypothesis concerns the anticipation of discharge prescriptions. Physicians may be reluctant to prescribe morphine in the ED if it is not included in the discharge prescription. There are no data to assess this decision criterion, even in outpatients. An NSAID or acetaminophen is regularly the most relevant option for continuing care in the outpatient, which is generally combined with targeted treatment in a multimodal approach (e.g., gabapentin for neuropathic pain, sumatriptan for headache, and alfuzosin for renal colic) (49, 72). However, few evidence-based plans are available regarding treatment plans from ED admission to discharge. Recently, the Australasian College for Emergency Medicine developed global recommendations for low back pain, both for analgesic management in the ED and for discharge planning (73).

The data suggest that education is another organizational factor associated with morphine prescribing. This association has been described previously (45–47). A moderate risk of IV morphine avoidance was observed among junior physicians (OR = 2.35). However, our ED organized twice-yearly training programs on opioids (74), and pain management support was available 24 h a day on the hospital intranet. Junior physicians may have preferred targeted practices to “one for all” IV morphine protocols and also modern concepts of individualized pain management. Most junior physicians were probably trained to use more non-opioid modalities. They may be more aware of the new robust alternatives and the specific patterns to consider, such as renal colic, minor trauma, or low back pain. Another hypothesis is that they have less confidence in VAS/NRS scores than their senior colleagues. It would have been interesting to compare junior and senior practices based on years of experience. In this study, pain patterns were not directly associated with the avoidance of IV morphine titration. This was an unexpected finding, especially for trauma (17, 62, 63) and visceral pain (75). The traumatological pattern in this study may have included a larger population than most studies, which are usually limited to minor trauma. Trends in univariate analyses were found for the visceral and urogenital patterns (*p* = 0.06), and a slightly weaker trend was found for the musculoskeletal pattern (*p* = 0.13). The weakness of these trends is probably explained by a lack of power in these subgroups. It would be interesting to evaluate the associations between some patterns highlighted in specific recommendations (72, 76) and ambulatory pathways in a larger population, for example, in a study designed to determine the crude or adjusted ORs for ambulatory renal colic or mild low back pain pathways. Complementary investigations could also evaluate the impact of these pain patterns on IV morphine prescribing, including physician grade, to assess this “modern education” hypothesis. For minor trauma, some authors have tested inhaled therapeutics (fentanyl and methoxyflurane) (77) in replacement of morphine titration, with success at the pharmacological level, but their organizational impact remains unknown (78). A comparison of these effects with the limitations of IV morphine is lacking. There are insufficient data to recommend these therapeutics as standard practice in ambulatory pathways. Physicians need specific methodologies to evaluate the implementation of these therapeutic innovations in the ED. Care pathways linking severe pain management and patient trajectories could be more relevant than other classic strategies but require evaluation.

Finally, NRS scores were significantly associated with the avoidance of the recommended IV morphine protocol. The pain intensity level was the only non-organizational factor involved. Moreover, an analysis of NRS intervals revealed a discrepancy between NRS levels at triage and the expected decision for protocol follow-up. Morphine was prescribed not only for very high pain levels (NRS scores of 10 and 9) but also for the lowest pain level (NRS 7). Adjusted ORs related to NRS scores were also inconsistent with respect to NRS intervals. These results suggest that the NRS score was not sufficient for automatic IV morphine management. Despite publications on the established reliability of NRS intervals (pain experience and clinical significance) (79), other reports question their relevance. Our results confirm these reports. One hypothesis is that physicians adjust VAS/NRS scores during triage to set priorities according to organizational constraints (80). This decision-making process is influenced by local processes and contextual factors. Care environment factors are likely to be involved in poor adherence to initial VAS/NRS-based protocols; specific evaluation methods are needed to highlight them (81) and confirm this hypothesis. A lack of additional scores to evaluate functional limitations due to pain could also be involved. Complementary tools such as the Behavioral Observation Scale 3 (BOS 3) (82) could be used to assess pain in ED patients. It was validated in 2017 as a rapid evaluation of non-sedated and non-geriatric adults. The BOS 3 is short and consists of five items (complaints, tense face, cautious movements, analgesic postures, and agitation/prostration), which provide a global score from 0 to 10. The patient is considered in pain if the total score is greater than or equal to 3. For ambulatory pathways, it would also be useful to evaluate the association between the severity of dysfunction and IV morphine prescription. Triggers for IV titration could be identified in this specific population.

This study had several limitations. First, it was conducted in a single center in an urban area with more severely ill patients, which introduces a selection bias. Nevertheless, the medical and paramedical staff of the ED discussed in this study is diverse and representative of ED caregivers from French hospitals. The activity level of this ED during the study period corresponded to a standard crowded period, and the total number of staff members by care area (physicians and nurses) was as expected, avoiding overcrowding bias (54). Thus, this study included a representative group eligible for IV morphine titration in the daily routine. This single-center design, in the highly standardized context of opioid management, allowed for a bedside study in real time, limiting data loss along care pathways. Conversely, the exhaustive nature of the recording of VAS/NRS scores, due to the computerized information system in the triage area, could explain the discrepancy regarding VAS/NRS intervals. An overassessment of pain, including patients who usually bear their pain and do not spontaneously express it at triage, could be involved. Other limitations were design related. The impact of socioeconomic parameters was not analyzed. Past medical history (including suspicion of hypersensitivity reactions) was not examined as a potential factor for the avoidance of IV morphine. The effects of pain management at home prior to ED admission, as well as pain relief after low protocol follow-up, were not investigated. This study of professional practices was not designed to evaluate oligoanalgesia as a potential outcome of IV morphine avoidance. This study focused on the organizational triggers for prescribing but not on patients’ feelings. Satisfaction with the treatment given, the desire for analgesics (83–85), or the overall effectiveness of the treatment were not assessed. The effect of not using IV morphine on the physician–patient relationship was not studied, although this is an important endpoint in all pain management. Conversely, the relationship between in-hospital prescriptions and home morphine savings was not explored. Although outpatients are known to be at high risk of opioid misuse (86), opioid prescriptions at discharge were not assessed in the ambulatory subgroup. Moreover, this case–control study lacks the power to assess the sparing of side effects with a restrictive morphine titration strategy (n = 46 in the morphine titration group). In a previous study of 621 patients, Lvovschi et al. (38) demonstrated the safety of the procedure. The incidence of all morphine-related adverse effects was low (10.8%; 95% CI, 8.6–13.5), and the incidence of severe respiratory adverse effects was 0 (95% CI, <0.6%). Furthermore, common analgesics (acetaminophen, NSAIDs, tramadol, nefopam, etc.) and co-analgesics (as antispasmodics) may be confounding factors involved in poor tolerability. No unusual adverse events were reported in either groups during the study period.

## 5 Conclusion

To conclude, in this bedside case–control study, adherence to a protocol based on IV morphine titration was significantly reduced in ambulatory pathways. The junior physician grade was also associated with this phenomenon. This study confirms the controversial relevance of pharmacologic management based only on NRS evaluation at triage. Multicenter evaluations are needed to test the hypotheses that these results are linked to a modern decision-making process; these results are explained by an organizational prioritization/downregulation of IV morphine in outpatients; and these results are robust in the population, including non-trauma patterns. In addition, it is important to evaluate patient satisfaction and the appropriateness of these new practices with analgesic desire.

The new goals of severe pain management in the ED should be to identify candidates for long ED stays who are eligible for first-line IV morphine and to consider alternative treatment for ambulatory patients. To avoid VAS/NRS misuse, ED caregivers need better tools and better pain education focused on ambulatory pathways.

## Data availability statement

The original contributions presented in the study are included in the article/Supplementary material; further inquiries can be directed to the corresponding author.

## Ethics statement

The studies involving humans were approved by the ethical committee of Rouen University Hospital (Review Board number E2019-27; approval date 4-12-2019). The studies were conducted in accordance with the local legislation and institutional requirements. The participants provided their written informed consent to participate in this study.

## Author contributions

VL: Conceptualization, Investigation, Methodology, Supervision, Writing – original draft, Writing – review & editing. FC: Writing – original draft, Writing – review & editing. KH: Data curation, Formal analysis, Investigation, Writing – review & editing. FL: Methodology, Writing – review & editing. L-MJ: Conceptualization, Methodology, Supervision, Writing – review & editing. M-PT: Conceptualization, Formal analysis, Investigation, Methodology, Supervision, Writing – review & editing.
